# Continuous sulfide supply enhanced autotrophic production of alcohols with *Clostridium ragsdalei*

**DOI:** 10.1186/s40643-022-00506-6

**Published:** 2022-03-03

**Authors:** Luis Oliveira, Simon Röhrenbach, Verena Holzmüller, Dirk Weuster-Botz

**Affiliations:** Department of Energy and Process Engineering, School of Engineering and Design, Chair of Biochemical Engineering, Technical University of Munich, Boltzmannstr. 15, 85748 Garching, Germany

**Keywords:** *Clostridium ragsdalei*, Syngas fermentation, Alcohol production, Sulfur limitation

## Abstract

Autotrophic syngas fermentation with clostridia enables the conversion of CO, CO_2_, and H_2_ into organic acids and alcohols. The batch process performance of *Clostridium ragsdalei* was studied in fully controlled and continuously gassed (600 mbar CO, 200 mbar H_2_, 200 mbar CO_2_) stirred-tank bioreactors. The final ethanol concentration varied as function of the reaction conditions. Decreasing the pH from pH 6.0–5.5 at a temperature of 37 °C increased the ethanol concentration from 2.33 g L^−1^ to 3.95 g L^−1^, whereas lowering the temperature from 37 to 32 °C at constant pH 6.0 resulted in a final ethanol concentration of 5.34 g L^−1^ after 5 days of batch operation. The sulphur availability was monitored by measuring the cysteine concentration in the medium and the H_2_S fraction in the exhaust gas. It was found that most of the initially added sulphur was stripped out within the first day of the batch process (first half of the exponential growth phase). A continuous sodium sulfide feed allowed ethanol concentrations to increase more than threefold to 7.67 g L^−1^ and the alcohol-to-acetate ratio to increase 43-fold to 17.71 g g^−1^.

**Graphical Abstract:**

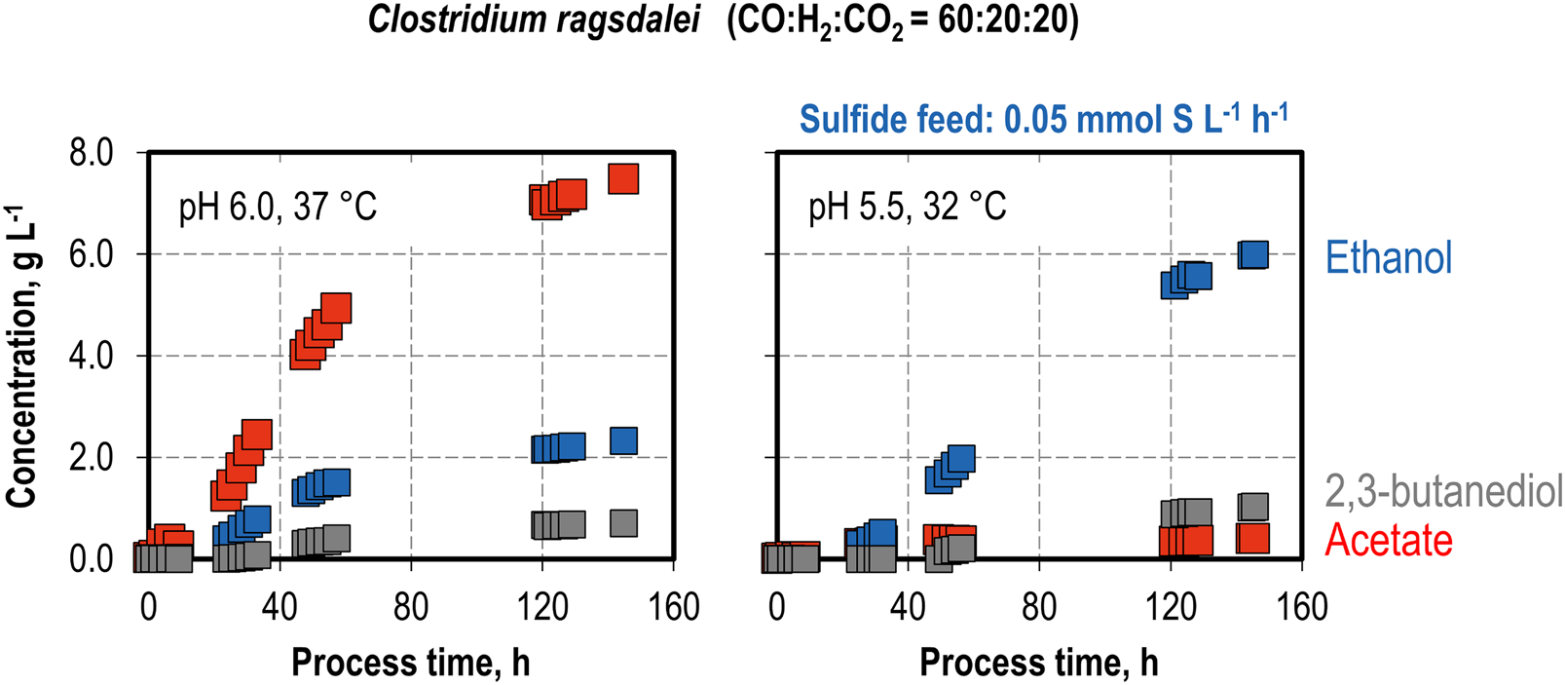

## Introduction

Syngas fermentation is a promising alternative for producing fuels and bulk chemicals from renewable resources, such as gasified biomass, and industrial exhaust gases, such as the exhaust from steel mills (Köpke et al. 2011; Liakakou et al. 2021). Syngas fermentation is a process competitive with the thermochemical conversion of syngas and offers the advantages of requiring milder temperatures and pressures, as well as being more tolerant to the ratio of H_2_ to CO and to possible impurities in the syngas like H_2_S and carbonyl sulfide (Griffin and Schultz 2012; Groher and Weuster-Botz 2016). Prominent representatives of the syngas-fermenting microorganisms include clostridia *C. ragsdalei*, *C. ljungdahlii*,* C. carboxidivorans*, and *C. autoethanogenum*. These are broadly studied acetogens which are able to conserve energy and fixate carbon in a non-cyclic metabolism from H_2_ and CO_2_ or CO.

These acetogens use the Wood–Ljungdahl pathway to produce acetyl-CoA through sequential reduction reactions, with CO or H_2_ serving as electron donors. Acetyl-CoA can be further converted to acetate with an ATP gain or other products like longer chained acids or their corresponding alcohols. Net ATP production is achieved through chemiosmosis driven by a membrane-bound Rnf complex, which pumps protons out of the cell by transferring electrons from ferredoxin to NAD, and a membrane-bound ATP synthase (Ramió-Pujol et al. 2015a; Schuchmann and Müller 2014).

*Clostridium ragsdalei *is an important acetogen that is able to convert CO, H_2_ and CO_2_ to ethanol, acetate, and 2,3-butanediol. *Clostridium ragsdalei *exhibits an optimal growth temperature of 37 °C (Bengelsdorf et al. 2018), optimal growth and ethanol production with CO as a substrate within pH 5.5–6.0, and it tolerates ethanol concentrations up to 30 g L^−1^ (Huhnke et al. 2010). *C. ragsdalei* possesses a genome-wide average nucleotide identity (ANI) with *C. autoethanogenum* and *C. ljungdahlii* of 95.9%, whereas the ANI between *C. ljungdhali* and *C. autoethanogenum* is 99.3% (Lee et al. 2019). Bengelsdorf et al. (2016) studied the genome of the clostridial strains *C. ljungdahlii*, *C. autoethanogenum*,* C. coskatii* and *C. ragsdalei*. *C. ragsdalei* shows some differences from the other three strains. Whereas these strains possess 20 genes encoding alcohol dehydrogenases, *C. ragsdalei* contains 16 genes. *C. ragsdalei* has four gene copies coding for aldehyde:ferredoxin oxidoreductases, whereas *C. ljungdahlii* and *C. autoethanogenum* have two gene copies coding for these enzymes. Bengelsdorf et al. (2016) also showed the performance in autotrophic batch processes in flasks with 50% (v/v) CO, 5% (v/v) CO_2_, 45% (v/v) H_2_ for these four strains, with *C. ragsdalei* producing comparable ethanol concentrations to *C. autoethanogenum*, whereas being the strain with the highest ethanol-to-acetate ratio and biomass-related ethanol yield.

CO-fermenting acetogens produce ethanol through the reduction of acetaldehyde, which can be formed from the direct reduction of acetyl-CoA, or the prior reduction of acetyl-CoA to acetate and subsequent reduction to acetaldehyde. The latter pathway has the energetic advantage of an ATP gain (Diender et al. 2015) and, with respect to *C. ljungdahlii,* has been shown to be preferable for use in ethanol production in syngas fermentation (Richter et al. 2016b).

Several aspects influence the autotrophic production of ethanol with acetogens, including medium composition, process temperature, and pH. Lowering the pH from the growth optimum is a broadly studied strategy used to increase ethanol production with clostridia (Abubackar et al. 2016; Infantes et al. 2020; Kundiyana et al. 2011; Richter et al. 2016a) because decreasing the pH triggers the shift from acetogenesis to solventogenesis (Ramió-Pujol et al. 2015a). At a lower pH, the extracellular concentration of undissociated acids is higher. The acids diffuse freely across the cell membrane and, in the case of acetic acid, promote ethanol formation (Richter et al. 2016b). If the pH drops below the optimum for solventogenesis, metabolic activities of the clostridia will be reduced considerably. For example, syngas uptake of *C. ljungdahlii* was interrupted in a batch process without pH control below pH 4.3 (Infantes et al. 2020).

Temperature and sulphur supply are less well studied as state variables in the fermentation of syngas with clostridia. Kundiyana et al. (2011) reported an increase in alcohol production to 1.89 g L^−1^ after 15 days in anaerobic flasks with *C. ragsdalei* (in the absence of a buffer upon reducing the temperature from 37 to 32 °C). This effect was related to the higher solubility of the gaseous substrates in the medium at lower temperatures. However, a lower temperature was investigated in conjunction with changes in pH and buffer concentrations, thus making it difficult to quantify the individual effect of temperature on the process. Ramió-Pujol et al. (2015b) observed higher ethanol productivity with *C. carboxidivorans* at 25 °C in anaerobic tubes as compared to 37 °C and partially related this improvement to a slowing of metabolic activity. Shen et al. (2020) showed by way of a comparative transcriptome analysis that *C. carboxidivorans* adapts to a decrease of temperature from 37 to 25 °C by up-regulating several pathways, e.g., the citrate cycle, butanoate metabolism, and the energy and amino acid metabolisms. Most of the published syngas fermentation processes were conducted at 37 °C.

Sulphur is a constituent of metal clusters in several enzymes present in the Wood–Ljungdahl pathway (Ragsdale 2008), so it plays a key role in autotrophic carbon fixation by acetogens. Sodium sulfide and cysteine are the most commonly used sources of sulphur in syngas fermentation with clostridia. In the case of cysteine, an L-cysteine desulfidase catalyzes the release of H_2_S from the thiol group (Gu et al. 2017), which can be further utilized. Few attempts have been made to improve sulphur availability in syngas fermentation with clostridia*.* Aklujkar et al. (2017) and Richter et al. (2016b) observed indications of a sulphur limitation in autotrophic processes with *C. ljungdahlii* through the upregulation of several genes related to the metabolism or uptake of sulphur-containing compounds. However, increasing the cysteine concentration had little effect on biomass growth and product formation with *C. autoethanogenum* and *C. ljungdahlii* (Abubackar et al. 2012; Infantes et al. 2020). No explanation for the ineffectiveness of this cysteine supplementation has yet been presented. Using *C. carboxidivorans* in batch syngas fermentations, Rückel et al. (2021) demonstrated considerable increases in the final ethanol and 1-butanol concentrations with the addition of 0.5 g L^−1^ or 1.0 g L^−1^ H_2_S added as thioacetamide, with a medium composition containing cysteine hydrochloride. This improvement in alcohol production was attributed to the additional sulphur availability.

We report herein on studies for improving ethanol production in continuously gassed (60% (v/v) CO, 20% (v/v) H_2_, and 20% (v/v) CO_2_) batch and fed-batch processes with *C. ragsdalei* in a controlled stirred-tank bioreactor. Temperature and pH were varied so as to observe their effect on biomass and product formation. Sulphur availability was studied based on the H_2_S fraction in the exhaust gas and the cysteine concentration in the medium. To the best of our knowledge, this is the first time that a more detailed information of sulphur availability has been presented regarding a syngas fermentation process with clostridia. Finally, the effects of a continuous sulphur supply and the combination thereof with the previously determined ideal temperature and pH were demonstrated in syngas fermentation with *C. ragsdalei*.

## Materials and methods

### Microorganism and growth conditions

*C. ragsdalei* (DSM 15248) was obtained from the German Collection of Microorganisms and Cell Cultures (DSMZ, Braunschweig, Germany). The medium contained 1 g L^−1^ yeast extract, mineral, trace elements, vitamins, and cysteine hydrochloride solutions. Details on the composition and preparation have been presented by Doll et al. (2018).

Precultures were prepared in 500-mL anaerobic flasks with 100 mL growth medium and 1.2 bar CO, 0.4 bar CO_2_, and 0.4 bar H_2_. The growth medium was previously anaerobized by boiling and subsequent N_2_ gassing. The precultures were kept in a shaking incubator (WiseCube WIS-20, Witeg Labortechnik GmbH, Wertheim, Germany) at 37 °C and 100 RPM, harvested after 44 h by centrifugation (10 min, 4500 RPM, Hettich Centrifuge, Rotixa 50 RS), and resuspended in anaerobic phosphate-buffered saline (PBS, pH 7.4).

### Stirred-tank bioreactor setup

The continuously gassed fermentation processes were conducted in a 2-L stirred-tank reactor (Infors HT, Bottmingen, Switzerland) with a working volume of 1 L, pH and temperature control and redox potential online measurement. The medium and stirred-tank bioreactor were previously sterilized (121 °C, 20 min), and the medium was transferred to the reactor using a peristaltic pump (Watson Marlow, Cornwall, England). The reactor was anaerobized through gassing with 5 NL h^−1^ of CO:CO_2_:H_2_ = 60:20:20% (v/v) for at least 12 h prior to inoculation. The sterile-filtered (0.2 μm cellulose filter, Chromafil RC20/15 MS, Macherey–Nagel GmbH & Co. KG, Düren, Germany) vitamin solution and the previously anaerobized and sterile cysteine hydrochloride stock solution were added to the reactor prior to inoculation as well. Two Rushton turbines were used for agitation at a stirrer speed of 800 RPM (volumetric power input of 3.5 W L^−1^); no overpressure was applied. The processes were inoculated with an initial optical density at 600 nm of 0.1, corresponding to an initial cell dry weight concentration of 0.042 g L^−1^.

In the processes in which the effect of different temperatures was investigated, temperatures were kept at 37 °C, 32 °C, and 27 °C at a pH of 6.0. In studies with a pH profile, the processes were performed at an initial pH of 6.0 without pH control until achieving the desired pH with a temperature of 37 °C. The pH were kept at pH 6.0, pH 5.5, and pH 5.0 by the addition of 3 M NaOH or 2 M H_2_SO_4_. In addition, an autotrophic batch process was studied without pH control.

In studies with the addition of sulphur, feed rates of 0.05 and 0.1 mmol S L^−1^ h^−1^ were employed using a peristaltic pump (Ismatec, Cole-Parmer GmbH, Wertheim, Germany). Stock solutions with 20 and 40 g L^−1^ Na_2_S 9 H_2_O, respectively, were used for the latter. In combination with a sulfide feed, the pH was controlled at either pH 6.0 or pH 5.5, and the temperature was set to either 37 °C or 32 °C.

All processes were gassed at a volume flow of 5 NL h^−1^ (0.083 vvm) and an inlet gas composition of CO:CO_2_:H_2_ = 60:20:20% (v/v), controlled by a mass flow controller (WMR 4000, Westphal Mess-und Regeltechnik GmbH, Ottobrunn, Germany). The exhaust gas was cooled to 2 °C with a reflux condenser and analyzed for CO, CO_2_, H_2_, and H_2_S, combining a mass flow meter (Wagner Mess- und Regeltechnik GmbH, Offenbach, Germany) and a micro-gas chromatograph (490 Micro GC System, Agilent Technologies, Santa Clara, USA).

### Analytical methods

Sterile sampling and the determination of the optical density OD_600_ as well as the product concentrations were processed as described in Doll et al. (2018). The linear correlation factor for the cell dry weight (CDW) concentrations and optical density at 600 nm (Genesys 10S UV–Vis; Thermo Scientific, Neuss, Germany) was 0.42 ± 0.03 g L^−1^. The acetate, ethanol, and 2,3-butanediol concentrations were measured by HPLC (Finnigan Surveyor, Thermo Fisher Scientific, Waltham, USA) with an ion exchange column (Aminex-HPX-87H, Biorad, Munich, Germany) and a refractive index detector (Finnigan Surveyor RI Plus Detector, Thermo Fisher Scientific, Waltham, USA). 5 mM H_2_SO_4_ was used as an eluent at 0.5 mL min^−1^ and a column temperature of 60 °C. The method used also enabled the detection of lactate and formate, but these were not observed in any of the processes being reported on.

The cysteine concentration was measured through the quantification of free thiols by way of a modification of the method based on Aitken and Learmonth (2009), in which the absorbance of the reacted sample at 412 nm is measured. Samples from the reactor were sterile-filtered and immediately analyzed with a reference. A new set of standards, which consisted of a growth medium with varying cysteine hydrochloride concentrations, was produced and measured for every process. 300 µL phosphate buffer, 300 µL diluted sample, and 12.5 µL Ellman’s solution were mixed and left to incubate for 5 min at room temperature before measurement. The phosphate buffer consisted of 33.79 g L^−1^ K_2_HPO_4_, 0.79 g L^−1^ KH_2_PO_4_, and 3.59 g L^−1^ EDTA dissolved in demineralized water. The Ellman’s solution consisted of 4 g L^−1^ 5,5′-dithiobis-(2-nitrobenzoic acid) dissolved in phosphate buffer and was stored at 8 °C to avoid degradation. A growth medium without cysteine hydrochloride was used for dilutions and as a reference. Measurements were conducted in duplicate.

### Bioprocess metrics

The maximum acetate, ethanol, and 2,3-butanediol production rates of the batch processes were estimated by a fitting approach. Firstly, concentrations of a component ${c}_{i}$ at a given time $t$ were interpolated with nonlinear regression using the following equation:

1 $${c}_{i}\left(t\right)={a}_{1, i}+\frac{{a}_{2, i}}{{a}_{3,i}\cdot {e}^{-{a}_{4,i}\cdot \left(t-{a}_{5, i}\right)}+{a}_{6, i}},$$

in which the parameters ${a}_{1:6, i}$ were identified for each process by minimizing the residual sum of squares (Microsoft Excel 2016, Microsoft, Redmond, Washington, USA).

Secondly, the production rate of a component ${r}_{i}$ was calculated with the derivative with time of the fitted concentrations, and the maximal production rate ${r}_{i, \mathrm{max}}$ was determined by setting the second derivative to zero:

2 $${r}_{i}=\frac{\mathrm{d}{c}_{i}}{\mathrm{dt}}; {r}_{i,\mathrm{ max}}={r}_{i}\left({t}_{i},\mathrm{ when }\frac{{\mathrm{d}}^{2}{c}_{i}}{{\mathrm{dt}}^{2}}=0\right).$$

## Results and discussion

### Effect of temperature and pH in the syngas fermentation

**Fig. 1:**
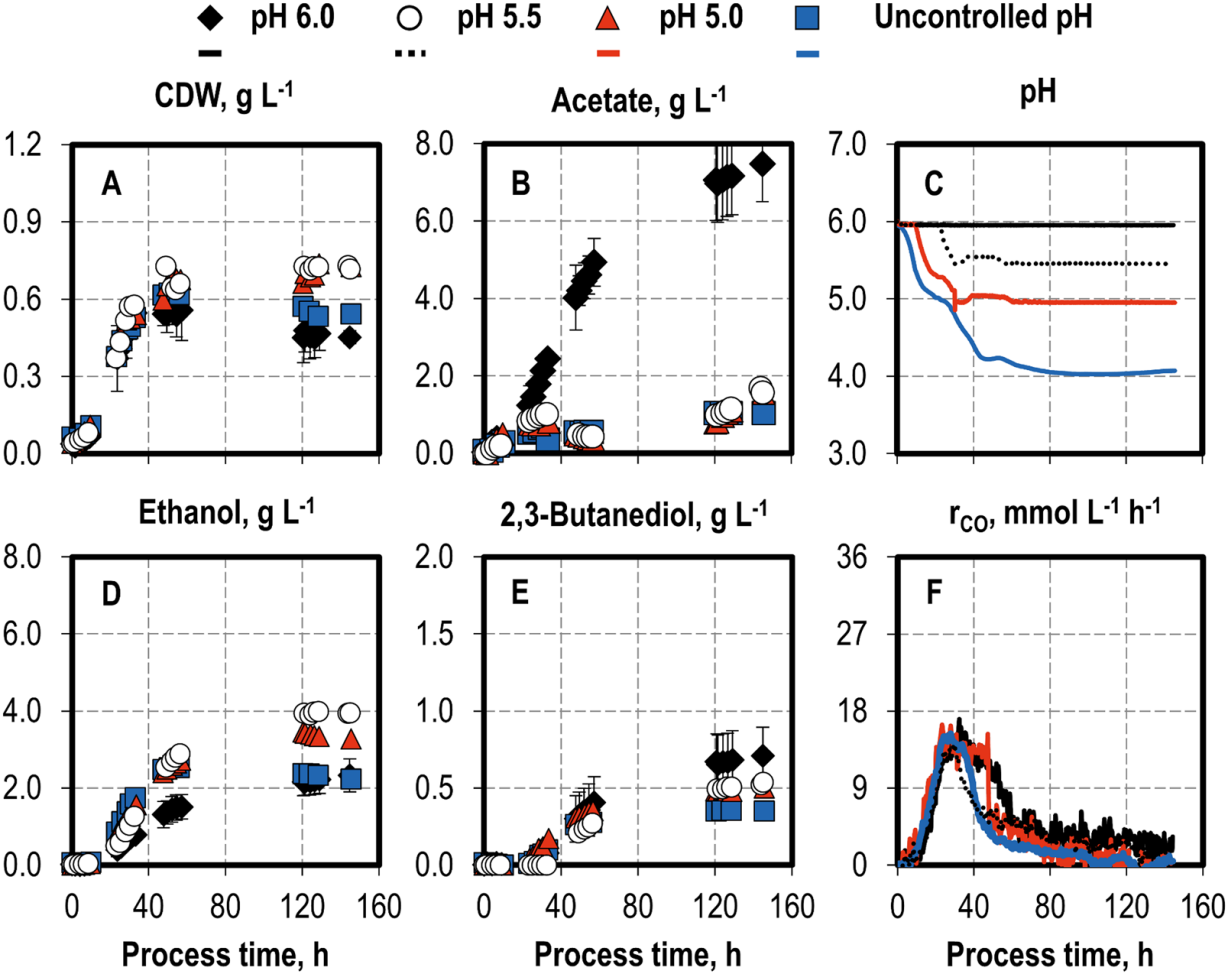
Variation of pH profiles in autotrophic batch processes with *C. ragsdalei* in a continuously gassed STR. Cell dry weight (**A** CDW) and product concentrations (**B** acetate; **D** ethanol; **E** 2,3-butanediol), pH (**C**) and CO uptake rates (**F** r_CO_): final pH 6 (black diamonds, black lines), pH 5.5 (white circles, dashed lines), pH 5.0 (red triangles, red lines), and uncontrolled pH (blue squares, blue lines). The batch processes were operated at a temperature of 37 °C, working volume of 1 L, volumetric power input of 3.5 W L^−1^, initial pH of pH 6.0, and a gas flow rate of 5 NL h^−1^ with inlet partial pressure of 0.2 bar H_2_, 0.2 bar CO_2_, and 0.6 bar CO. Standard deviations are shown for the processes at pH 6.0, which were conducted in triplicate; CO uptake rates are shown without standard deviations

**Fig. 2:**
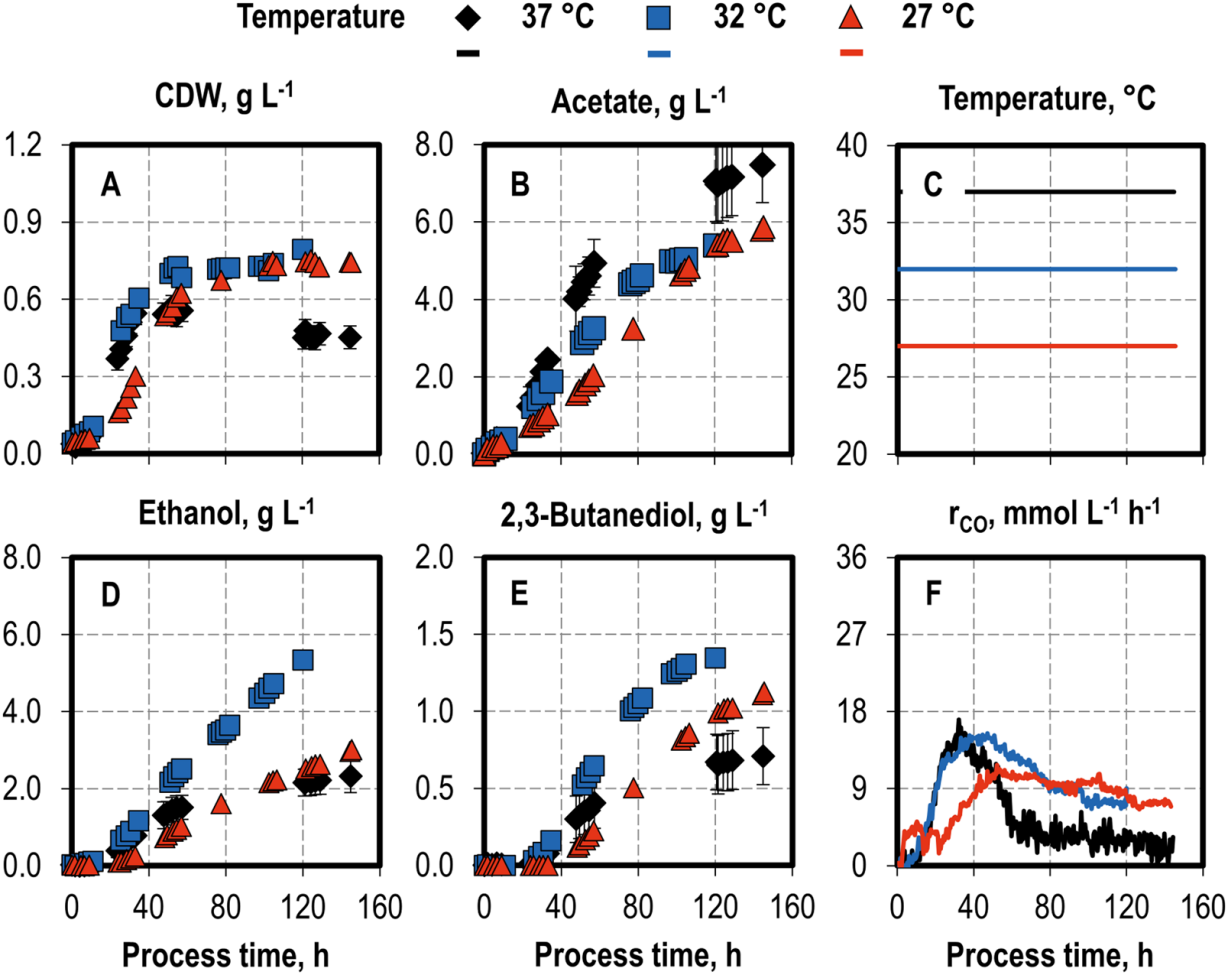
Variation of temperatures in autotrophic batch processes with *C. ragsdalei* in a continuously gassed STR. Cell dry weight (**A** CDW) and product concentrations (**B** acetate; **D** ethanol; **E** 2,3-butanediol) and the CO uptake rates (**F** r_CO_): 37 °C (**C** black diamonds, black lines), 32 °C (**C** blue squares, blue lines) and 27 °C (**C** red triangles, red lines). The batch processes were operated at a working volume of 1 L, volumetric power input of 3.5 W L^−1^, pH 6.0, and a gas flow rate of 5 NL h^−1^ with an inlet partial pressure of 0.2 bar H_2_, 0.2 bar CO_2_, and 0.6 bar CO. Standard deviations are shown for the processes at pH 6.0, which were conducted in triplicate; CO uptake rates are shown without standard deviations

Continuously gassed batch processes with *C. ragsdalei* were conducted separately, with varying pH profiles (Fig. 1) and temperatures (Fig. 2). In addition, the biomass and product formation, as well as the pH and CO uptake rates, was monitored. The pH stabilized after 35 h in the batch processes with a final control of pH 5.0 and pH 5.5, respectively. The batch process without a pH control resulted in pH 4.0 after 80 h.

The final CDW concentration differed strongly with varying pH profiles, ranging from 0.45 g L^−1^ at pH 6.0 to 0.73 g L^−1^ at pH 5.5. It is noteworthy that the CDW concentration decreased in the processes with pH 6.0 and uncontrolled pH after 50 h, but this CDW concentration decrease was absent in the processes at pH 5.0 and pH 5.5. Varying the pH profiles had little influence on the CO uptake rate in the first 35 h, achieving roughly 15 mmol L^−1^ h^−1^ in all batch processes, with a higher pH maintaining CO uptake rates at a higher level for a longer period. This implies that, during the growth phase, a pH as low as pH 4.5 (seen in the process without pH control) had little effect on biomass growth and CO uptake. The total CO uptake (data not shown) decreased with decreasing final pH.

Lowering the pH had a strong effect on acetate concentration, which decreased from 7.48 g L^−1^ at pH 6.0 to less than 1.6 g L^−1^ acetate in the other batch processes. In the processes with a final pH 5.5 or pH 5.0, a decrease in the acetate concentration was observed between hours 30 and 50, indicating a net uptake of acetate for ethanol production. Ethanol production began after hour 20 and was observed in both the growth and stationary phases. The highest ethanol concentration, of 3.95 g L^−1^, was reached at pH 5.5, followed by 3.27 g L^−1^ at pH 5.0. Clearly, a lower pH shifted carbon fixation from acetate to ethanol and concomitantly reduced total carbon fixation. This shift from acetate to ethanol production has been reported previously. Lowering the pH, from pH 5.75–4.75, in batch syngas fermentations in anaerobic flasks with *C. autoethanogenum* had a positive effect on ethanol production, but negatively impacted biomass formation, and had no effect on acetate production (Abubackar et al. 2012). This positive effect of a lower pH on ethanol formation was exploited in a two-stage setup with a CSTR and a continuously operated bubble column reactor with *C. ljungdahlii*, achieving an ethanol concentration of 19.7 g L^−1^ (428.4 mM) and an alcohol-to-acetate ratio of 2.34 g g^−1^ (Richter et al. 2013).

The 2,3-butanediol concentration also correlated with the pH, decreasing from 0.71 g L^−1^ at pH 6.0 to 0.35 g L^−1^ at an uncontrolled pH. This is the first time that the negative effect of a lower pH on the 2,3-butanediol concentration has been demonstrated in the CO fermentation. Köpke et al. (2011) hypothesized that the 2,3-butanediol production was triggered to balance intracellular reducing agents and counteract acidification by pyruvate production because the 2,3-butanediol formation resumed at a lower pH. However, the results of the present study contradict this observation. It is possible that the link lies in the availability of reducing agents. Given that 2,3-butanediol is formed from pyruvate by the reduction of acetoin with NADPH (Köpke et al. 2014; Zhu et al. 2020), its formation might be limited by a limited amount of reducing agents and their redirection to acetate reduction to ethanol promoted by lower pH levels.

The highest maximum ethanol production rate of 1.97 g L^−1^ day^−1^ was observed at pH 5.5—more than twice that at pH 6.0 (data not graphically shown). In fact, all of the processes at a lower pH increased this rate. Overall, a pH of 5.5 showed several benefits, including increasing ethanol concentrations and maximum production rates as well as shifting the carbon fixation from acetate production to that of ethanol. Moreover, the final CDW concentration also increased, whereas no strong decrease in the maximal CO uptake rate was observed.

Temperatures of 32 °C and 27 °C resulted in higher final CDW concentrations than at 37 °C. Acetate formation was reduced in both cases, from 7.48 g L^−1^ at 37 °C to 5.40 g L^−1^ at 32 °C. Production of both alcohols increased, with a maximum ethanol concentration of 5.34 g L^−1^ and 2,3-butanediol concentration of 1.35 g L^−1^ at 32 °C, which were the highest ethanol and 2,3-butanediol concentrations in this single-parameter analysis. The maximum CO uptake rate remained unchanged at 32 °C, but decreased to 10 mmol L^−1^ h^−1^ at 27 °C. The decrease in CO uptake rates after peaking was less accentuated for the lower temperatures.

Lowering the process temperature to 32 °C showed strong advantages in comparison to the standard autotrophic batch process at 37 °C. It increased both the final ethanol concentration and its maximal production rate, as well as the final 2,3-butanediol concentration and the alcohol-to-acetate ratio. This improved alcohol production is related to the slower metabolic rate of the cells at a lower temperature, which helps to prevent an “acid crash” (Ramió-Pujol et al. 2015b). The relevant effect is the suppression of solventogenesis, which prevents the production of alcohols by acetogens, despite the presence of external triggers (Maddox et al. 2000). “Acid crash” has been associated with the external concentration of undissociated acids (Maddox et al. 2000) as well as the intracellular concentration of formate (Wang et al. 2011) and is distinct from acidogenic fermentation, which is the metabolic regulated production of acids by acetogens at a higher pH. Indeed, in this single-parameter study lower acetate production rates were observed in all batch processes as compared to the standard at 37 °C and pH 6.0 (2.76 g L^−1^ d^−1^). The second highest maximum acetate production rate was observed at 32 °C with 1.65 g L^−1^ d^−1^, corresponding to a 40.2% decrease.

In addition to the “acid crash”, the extracellular acetate concentration controls carbon and redox metabolism to maintain ATP homeostasis, as proposed by Valgepea et al. (2017) based on results obtained with *C. autoethanogenum*. Higher extracellular acetate levels partially uncouple the proton motive force (PMF) from ATP synthesis and increase the ATP maintenance cost. This forces a redirection of carbon from biomass to product formation. Excessive acetate production, as observed at pH 6.0, would thus combine higher ATP maintenance costs, a PMF uncoupled from ATP synthesis, and the “acid crash”. The latter would favor further acetate production and reinforce the other two effects. This is consistent with the low alcohol and biomass production observed at pH 6.0.

A strong improvement in alcohol production was observed at both pH 5.5 and a temperature of 32 °C. A lower pH considerably increased the alcohol-to-acetate ratio (2.84 g g^−1^) through metabolic regulation, but this advantage was offset by lower CO uptake rates in the stationary phase and a lower final 2,3-butanediol concentration. In turn, the process temperature of 32 °C combined higher alcohol production with a higher total CO uptake, thus advantageously promoting 2,3-butanediol formation. This was achieved at an alcohol-to-acetate ratio of 1.23 g g^−1^ and a final acetate concentration of 5.40 g L^−1^.

### Sulphur availability and supply

**Fig. 3:**
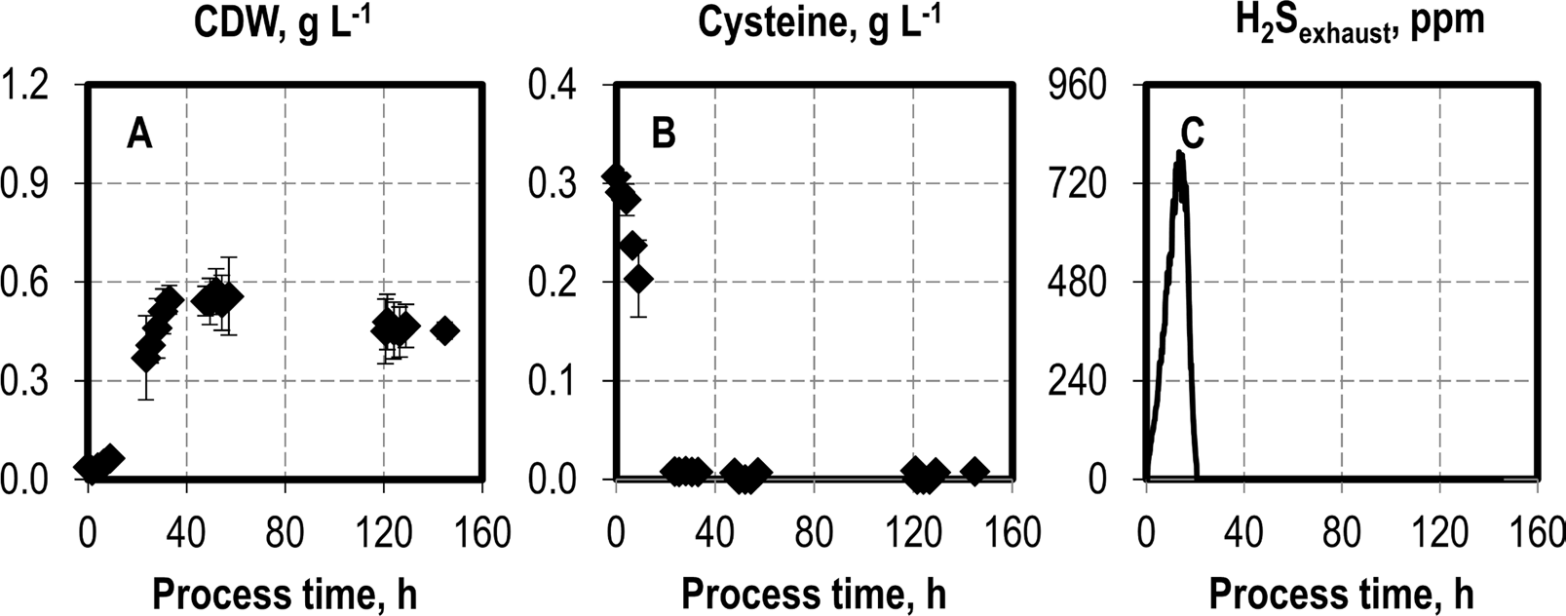
Sulphur availability in autotrophic batch process with *C. ragsdalei* in a continuously gassed STR: cell dry weight (**A** CDW) concentration, cysteine concentration (**B**), and hydrogen sulfide in exhaust gas (**C**). The batch processes were operated at a working volume of 1 L, volumetric power input of 3.5 W L^−1^, pH 6.0, and a gas flow rate of 5 NL h^−1^ with an inlet partial pressure of 0.2 bar H_2_, 0.2 bar CO_2_, and 0.6 bar CO. The batch process was conducted in triplicate; H_2_S in the exhaust is shown without standard deviations

There is an apparent contradiction in the published results regarding sulphur use. *C. ljungdahlii* was sulphur-limited in the syngas fermentation with cysteine (Richter et al. 2016b), but increasing the cysteine concentrations did not have a considerable effect on biomass growth and alcohol production (Infantes et al. 2020). To clarify this, the sulphur availability in an autotrophic batch process with *C. ragsdalei* at pH 6.0 and 37 °C was monitored using the concentration of cysteine in the medium and the H_2_S fraction in the exhaust gas (Fig. 3).

The cysteine concentration decreased from the initial 307.17 ± 5.24–7.85 ± 0.18 mg L^−1^ within the first 23 h of the process, indicating that most of the cysteine was converted within the first half of the exponential growth phase at an average volumetric cysteine conversion rate of 0.108 mmol L^−1^ h^−1^. At the same time, up to 795 ppm H_2_S was detected in the exhaust gas. Out of the initially available 2.55 mM sulphur introduced into the medium as cysteine, 1.8 mM was stripped by the exhaust gas as H_2_S, representing a measured loss of 74% (n/n). *C. ragsdalei* accesses the sulphur in cysteine by releasing the highly volatile H_2_S, which is quickly stripped from the continuously gassed reactor, an effect clearly seen in the process presented. This high rate of H_2_S stripping from a gassed STR was also demonstrated by Hu et al. (2011), who reported the nearly complete stripping of 0.8 mM sulfide within 150 min after the addition of sodium sulfide in a 1-L stirred-tank reactor at 37 °C.

Despite this rapid conversion of cysteine and the stripping of hydrogen sulfide within the first 24 h, biomass growth continued until hour 40, and production formation occurred until the process end, with an according CO uptake. We hypothesize that further biomass growth may be supported by sulphur sources in the yeast extract, but these were not detected by the methods used. In turn, product formation can occur in a manner uncoupled from biomass growth, as has also been shown in autotrophic fed-batch processes with *C. carboxidivorans* at CO partial pressures up to 1.05 atm (Hurst and Lewis 2010) and in autotrophic fed-batch processes with *C. ljungdahlii* (Hermann et al. 2020).

This is the first time that the use of cysteine in syngas fermentation has been monitored, and the results showed that most of the available sulphur as cysteine was lost before the end of the exponential growth phase by gas stripping. This sulphur loss is one possible explanation for the ineffectiveness of increasing the cysteine concentration in the syngas fermentation with *C. autoethanogenum* and *C. ljungdahlii* as reported by Abubackar et al. (2012) and Infantes et al. (2020), respectively.

**Fig. 4:**
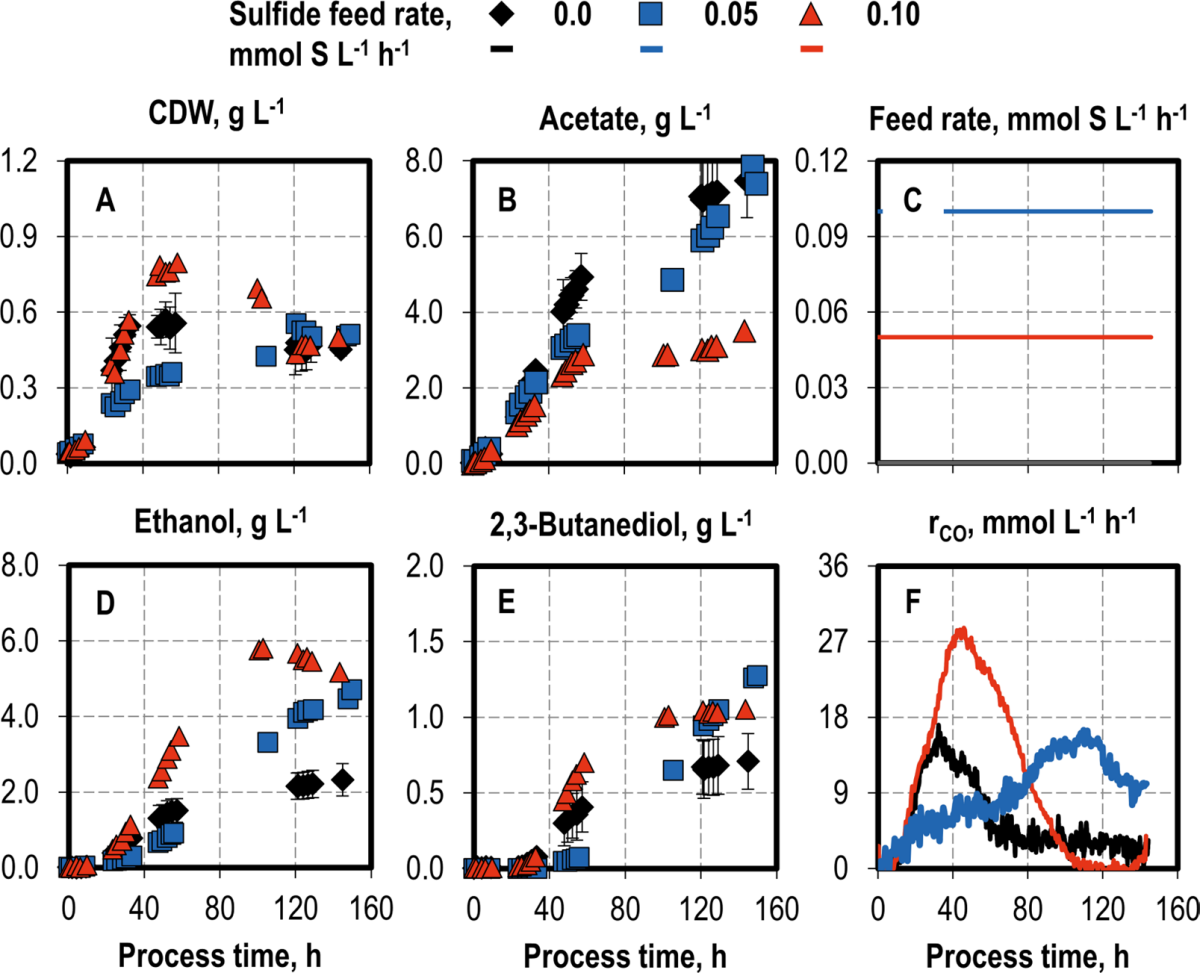
Variation of sulfide feeding in autotrophic fed-batch processes with *C. ragsdalei* in a continuously gassed STR. Cell dry weight (**A** CDW) and product concentrations (**B** acetate; **D** ethanol; **E** 2,3-butanediol) and CO uptake rates (**F** r_CO_): no feed (**C** black diamonds, black lines), 0.1 mmol S L^−1^ h^−1^ (**C** blue squares, blue lines) and 0.05 mmol S L^−1^ h^−1^ (**C** red triangles, red lines). The fed-batch processes were operated at a temperature of 37 °C, working volume of 1 L, volumetric power input of 3.5 W L^−1^, pH 6.0, and a gas flow rate of 5 NL h^−1^ with an inlet partial pressure of 0.2 bar H_2_, 0.2 bar CO_2_, and 0.6 bar CO. Standard deviations are shown for the processes at pH 6.0, which were conducted in triplicate; CO uptake rates are shown without standard deviations

The rapid stripping of H_2_S is an inherent disadvantage of using cysteine as a sulphur source in continuously gassed batch processes, since it limits the available sulphur. To address this limitation, the processes were conducted via the continuous addition of sulfide at feed rates of 0.05 mmol S L^−1^ h^−1^ and 0.1 mmol S L^−1^ h^−1^, with the latter rate roughly corresponding to the rate of cysteine conversion observed in the reference within the first 23 h (Fig. 4). Despite very similar final CDW concentrations, the supply of 0.1 mmol S L^−1^ h^−1^ slowed biomass formation, whereas the supply of 0.05 mmol S L^−1^ h^−1^ strongly promoted it in the first 60 h of the fed-batch process.

The fed-batch process with the sulphur feed of 0.05 mmol S L^−1^ h^−1^ can be divided into three phases. First, an initial phase of biomass production (0–50 h), which was coupled with acetate and ethanol formation and ended with the maximum CO uptake rate. Second, a phase of biomass loss (50–120 h), during which the CDW concentration decreased, acetate production stopped, and alcohol production occurred at lower rates, with the ethanol concentration reaching a maximum of 5.80 g L^−1^ at a process time of 102 h. Third, a stationary phase (120–144 h), in which the CDW concentration stabilized and acetate formation resumed, whereas ethanol concentration decreased and 2,3-butanediol concentration remained unchanged.

The extra addition of sulfide increased the final alcohol concentration at both of the feed rates studied, even though the final CDW concentrations were similar to the batch process without a sulfide feed. Ethanol formation occurred in a manner both coupled with and decoupled from biomass growth, whereas 2,3-butanediol production was exclusively decoupled from growth. This suggests that the positive effect of sulphur exceeds that of growth limitation. Na_2_S is a known reducing agent, but the culture redox potential (CRP) measurements showed no clear trend, with the minimum CRP achieved in the process without a sulfide feed (data not shown). H_2_S sulfide can freely diffuse into the cell (Ntagia et al. 2020), possibly altering intracellular conditions. However, the data acquired in this work have offered no insights into the possible biological reasons for this shift to alcohol production.

The maximum CO uptake rate increased considerably to 28 mmol L^−1^ h^−1^ at the lower sulfide feed rate (0.05 mmol S L^−1^ h^−1^), whereas the CO uptake peaked at a higher feed rate (0.1 mmol S L^−1^ h^−1^) only after process hour 100, indicating sulfide inhibition.

The sulphur quantities added over 144 h were 7.2 mmol S, and 14.4 mmol S at the feed rates of 0.05 mmol S L^−1^ h^−1^ and of 0.1 mmol S L^−1^ h^−1^, respectively. Accordingly, H_2_S was detected in the exhaust gas at amounts of 5.43 mmol, and 12.12 mmol for the feed rates of 0.05 mmol S L^−1^ h^−1^_,_ and of 0.1 mmol S L^−1^ h^−1^, corresponding to 56%, and 72% (n/n) of the available sulphur supplied as cysteine and sodium sulfide, respectively (data not graphically shown). Out of the sulphur supplied as sodium sulfide and cysteine, 37% and 24%, respectively, was missing when considering the final free thiol concentrations in these processes. Therefore, the form in which the missing sulphur was present in the medium remains an open question. We hypothesize that, given higher sulfide availability, insoluble sulfides such as ZnS and NiS were formed, which in turn were not detected with the method used for the analysis of free thiols.

The continuous sulfide feed presented herein provides an alternative for increasing sulphur availability in continuously gassed syngas fermentations, which can be inherently sulphur-limited. The addition of 0.05 mmol S L^−1^ h^−1^ promoted biomass growth, increased the total final alcohol concentration by more than twofold (5.18 g L^−1^ ethanol and 1.05 g L^−1^ 2,3-butanediol), raised the alcohol-to-acetate ratio by fourfold (1.77 g g^−1^), and nearly doubled the maximum CO uptake rate (28 mmol L^−1^ h^−1^) compared to the process without sulfide addition. The maximum ethanol productivity was also increased by threefold, to 2.61 g L^−1^ day^−1^, which was the highest value obtained in this study. This indicates that the sulfide feed is an effective strategy for improving alcohol productivity in syngas fermentation. Nevertheless, since the biological mechanisms through which the sulfide feed could promote alcohol formation are unknown, tackling this topic by identifying which genes and pathways are changed with the sulfide feed may give clues for strain improvement.

Moreover, the results show that the use of cysteine as main sulphur source has the inherent advantage of insufficiently providing the process with usable sulphur, thus limiting alcohol production. Since cysteine is also a costly component, further research in partially or completely replacing cysteine in the syngas fermentation with an appropriate sulfide feed could lower the costs of ethanol and 2,3-butanediol production.

### Combining lower pH and temperature with sulfide feed

**Fig. 5:**
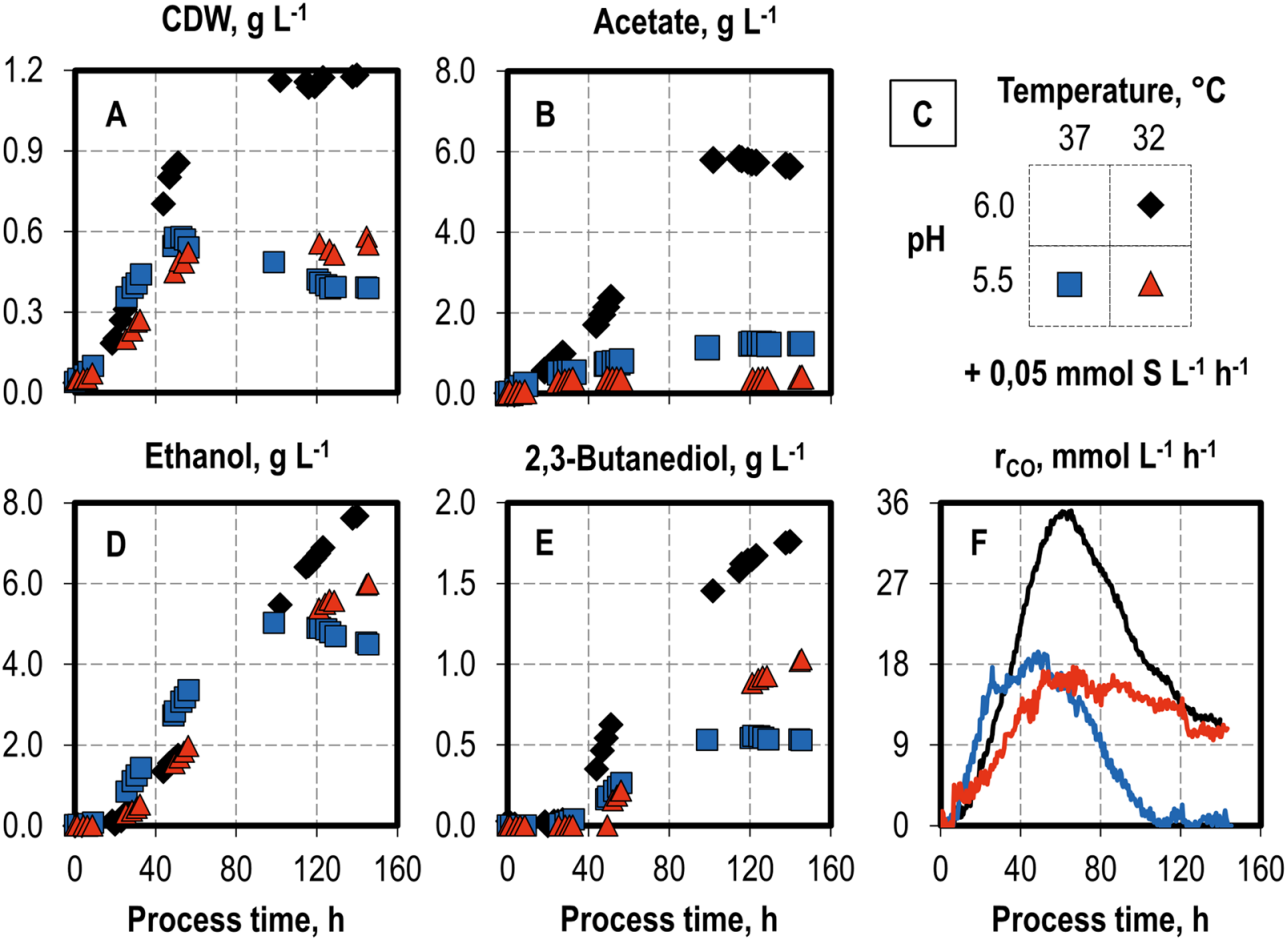
Variation of pH profiles and temperatures in autotrophic sulfide fed-batch processes with *C. ragsdalei* in a continuously gassed STR. Cell dry weight (**A** CDW) and product concentrations (**B** acetate; **D** ethanol; **E** 2,3-butanediol) as well as CO uptake rates (**F** r_CO_). Parameter combinations are shown in **C**: pH 6.0 and 32 °C (black diamonds, black lines), pH 5.5 and 37 °C (blue squares, blue lines) and pH 5.5 and 32 °C (red triangles, red lines). The fed-batch processes were operated at a Na_2_S 9 H_2_O feed rate of 0.05 mmol L^−1^ h^−1^, working volume of 1 L, volumetric power input of 3.5 W L^−1^, gas flow rate of 5 NL h^−1^ and an inlet partial pressure of 0.2 bar H_2_, 0.2 bar CO_2_, and 0.6 bar CO

After the positive effects of a 0.05 mmol S L^−1^ h^−1^ sulfide feed had been demonstrated in the syngas fermentation, a further increase in alcohol productivity was attempted by coupling the sulfide feed and the previously investigated state variables of temperature and pH. Since a temperature of 32 °C and pH 5.5 provided positive results in the single-parameter analysis, these state variables were both individually combined with the sulfide feed used simultaneously (Fig. 5).

Combining the sulfide feed at pH 5.5 led to a final CDW concentration of 0.39 g L^−1^, with a maximum of 0.58 g L^−1^ being reached at 50 h. No product was formed after hour 100, which was related to the interruption of CO uptake. The final ethanol and 2,3-butanediol concentrations were 4.49 g L^−1^ and 0.53 g L^−1^, respectively. Thus, a higher ethanol concentration was observed in comparison to the analogous process without a sulfide feed (3.95 g L^−1^). Given the lower acetate production, the alcohol-to-acetate ratio increased to 4.05 g g^−1^, an increase of 42%. The final concentrations of all products decreased in this process in relation to other processes with a sulfide feed rate of 0.05 mmol S L^−1^ h^−1^, reflecting the lower total carbon fixation at a lower pH already observed in the single-parameter studies.

The combination of 32 °C and the sulfide feed prevented the CDW concentration from dropping after the peak and promoted further biomass growth, resulting in a final, and the highest, CDW concentration of 1.18 g L^−1^. This represents an almost threefold increase in comparison to the batch process without sulfide addition at pH 6.0 and 37 °C. The maximum CO uptake rate was increased to 34.7 mmol L^−1^ h^−1^, and the final ethanol and 2,3-butanediol concentrations increased to 7.67 g L^−1^ and 1.76 g L^−1^, respectively. The biomass-related yields of ethanol (6.68 g g^−1^) and 2,3-butanediol (1.51 g g^−1^) decreased by 5.4% and 15.5%, respectively, compared to the analogous batch process without a sulfide feed. However, given the considerably higher final CDW concentration, the benefits of the sulfide feed clearly outweigh the milder decrease in alcohol biomass-related yields.

The sulfide feed at pH 5.5 and 32 °C resulted in clear improvements over pH 5.5 and 37 °C. A higher final CDW concentration was achieved, and the final ethanol concentration increased from 4.49 g L^−1^ to 6.00 g L^−1^. However, based on the biomass produced, the ethanol yield slightly decreased, from 12.57 g g^−1^ to 11.79 g g^−1^. Further benefits of the sulfide feed at pH 5.5 and 32 °C included CO uptake as well as ethanol and 2,3-butanediol formation occurring throughout the whole process length. It is noteworthy that these process conditions clearly suppressed acetate formation and shifted carbon almost completely to alcohol formation. This is emphasized by the high alcohol-to-acetate ratio under these conditions (17.71 g g^−1^), which is, to the best of our knowledge, the highest ever obtained in the published results for syngas fermentation with *C. ragsdalei*.

Surprisingly, with a sulfide feed of 0.05 mmol L^−1^ h^−1^ at pH 5.5 and 32 °C, the 2,3-butanediol concentration almost doubled to 1.03 g L^−1^, in comparison to the process with the same sulfide feed and pH at 37 °C. In one respect, this can be attributed to the 42% increase in final CDW concentration at 32 °C over that at 37 °C and, in another respect, to the cell’s ability to take up CO for a longer time period at a lower temperature.

The results achieved in this work with *C. ragsdalei* regarding ethanol space–time yields and ethanol-to-acetate ratios clearly demonstrate the benefits of a sulfide feed in combination with a lower temperature and lower pH, as shown in comparison with published results (Table 1). Devarapalli et al. (2016) obtained in a semi-continuous trickled bed reactor a maximal estimated ethanol space–time yield of 30.41 mg L^−1^ h^−1^, whereas the highest space–time yield obtained in this work was 54.82 mg L^−1^ h^−1^ at pH 6.0 with 32 °C and a sulfide feed of 0.05 mmol S L^−1^ h^−1^, an improvement of 80.3%. Kundiyana et al. (2010) reported an ethanol-to-acetate ratio of 5.24 g g^−1^, whereas at pH 5.5 with 32 °C and a sulfide feed of 0.05 mmol S L^−1^ h^−1^, an ethanol-to-acetate ratio of 14.82 g g^−1^ was obtained, an improvement of 183%. Further increase in the ethanol productivities might be achieved by an alternative operation mode which allows for higher cell densities, such as a continuous operation mode with cell retention, and should be subject of further research.

**Table 1:** Comparison of final ethanol concentrations, total ethanol space–time yields, and ethanol-to-acetate ratio in autotrophic processes with *C. ragsdalei*

Reactor mode Gas composition CO/H_2_/CO_2_/N_2_, % (v/v)	Process time, h Temperature, °C pH Medium additive	Ethanol concentration, g L^−1^ Ethanol space–time yield, mg L^−1^ h^−1^ Ethanol to acetate, g g^−1^	References
Fed-batch CSTR, uncontrolled pH 20/5/15/60	360 h; 37 °C; pH 6.1 1 g L^−1^ YE^a^	6.10 g L^−1^; 16.94 mg L^−1^ h^−1^; 4.07 g g^−1^	Maddipati et al. (2011)
	360 h; 37 °C; pH 6.1 10 g L^−1^ CSL^b^	8.60 g L^−1^; 23.89 mg L^−1^ h^−1^; 3.58 g g^−1^	
	360 h; 37 °C; pH 6.1 20 g L^−1^ CSL	9.60 g L^−1^; 26.67 mg L^−1^ h^−1^; 2.82 g g^−1^	
	1416 h; 37 °C; pH 6.0 10 g L^−1^ CSL	25.26 g L^−1^; 17.84 mg L^−1^ h^−1^; 5.24 g g^−1^	Kundiyana et al. (2010)
Semi-continuous trickle bed reactor, uncontrolled pH 38/28.5/28.5/5	171 h; 37 °C; pH 5.6 0.5 g L^−1^ YE	5.70 g L^−1^; 30.41 mg L^−1^ h^−1^ (estimated); 0.46 g g^−1^	Devarapalli et al. (2016)
Fed-batch CSTR, controlled pH *Sulfide feed 0.05 mmol S L^−1^ h^−1^ 60/20/20/0	144 h; 37 °C; pH 6.0 1 g L^−1^ YE	2.33 g L^−1^; 16.18 mg L^−1^ h^−1^; 0.31 g g^−1^	This study
	*144 h; 37 °C; pH 6.0 1 g L^−1^ YE	5.18 g L^−1^; 35.94 mg L^−1^ h^−1^; 1.48 g g^−1^	
	*140 h; 32 °C; pH 6.0 1 g L^−1^ YE	7.67 g L^−1^; 54.82 mg L^−1^ h^−1^; 1.36 g g^−1^	
	*144 h; 32 °C; pH 5.5 1 g L^−1^ YE	6.00 g L^−1^; 41.65 mg L^−1^ h^−1^; 14.82 g g^−1^	

^a^Yeast extract

^b^Cotton seed liquor

## Conclusion

Sulphur is a critical component in the batch performance of syngas fermentation with *C. ragsdalei* and represents an inherently limiting factor in processes with cysteine as a sulphur source and reducing agent. This was successfully demonstrated in this work both by quantifying the sulphur loss in the exhaust gas and by the improved fed-batch process performance with respect to alcohol formation upon continuously feeding sodium sulfide. The results presented herein pave the way for an improved syngas fermentation process design, since high alcohol production with negligible acetate formation could be achieved by implementing sulfide feed at a decreased temperature and pH. Further research into the fine-tuning of temperature, pH, and sulfide feed rates as well as into the shift to a continuous operation mode and the replacement of cysteine in the medium composition could lead to higher alcohol productivities, thus increasing the economic viability of syngas fermentation. Furthermore, the identification of which genes and pathways were altered under the sulfide feed with a comparative transcriptomic analysis could elucidate the mechanisms with which the sulfide feed promoted alcohol formation.

## Data Availability

The authors can confirm that all relevant data are included in the article and/or its Additional files.

## Abbreviations

ANI: Average nucleotide identity
ATP: Adenosine triphosphate
CDW: Cell dry weight
NAD: Nicotinamide adenine dinucleotide
STR: Stirred-tank reactor
WLP: Wood–Ljungdahl pathway
